# Perfusion-based co-culture model system for bone tissue engineering

**DOI:** 10.3934/bioeng.2020009

**Published:** 2020-05-29

**Authors:** Stephen W. Sawyer, Kairui Zhang, Jason A. Horton, Pranav Soman

**Affiliations:** 1 Department of Biomedical and Chemical Engineering, Syracuse University, Syracuse, NY, USA; 2 Department of Orthopedic Surgery, SUNY Upstate Medical University, Syracuse, NY, USA

**Keywords:** Gelatin methacrylate, mineral formation, cell encapsulation, perfusion, bioreactor, endothelial cells

## Abstract

In this work, we report on a perfusion-based co-culture system that could be used for bone tissue engineering applications. The model system is created using a combination of Primary Human Umbilical Vein Endothelial Cells (HUVECs) and osteoblast-like Saos-2 cells encapsulated within a Gelatin Methacrylate (GelMA)-collagen hydrogel blend contained within 3D printed, perfusable constructs. The constructs contain dual channels, within a custom-built bioreactor, that were perfused with osteogenic media for up to two weeks in order to induce mineral deposition. Mineral deposition in constructs containing only HUVECs, only Saos-2 cells, or a combination thereof was quantified by microCT to determine if the combination of endothelial cells and bone-like cells increased mineral deposition. Histological and fluorescent staining was used to verify mineral deposition and cellular function both along and between the perfused channels. While there was not a quantifiable difference in the amount of mineral deposited in Saos-2 only versus Saos-2 plus HUVEC samples, the location of the deposited mineral differed dramatically between the groups and indicated that the addition of HUVECs within the GelMA matrix allowed Saos-2 cells, in diffusion limited regions of the construct, to deposit bone mineral. This work serves as a model on how to create perfusable bone tissue engineering constructs using a combination of 3D printing and cellular co-cultures.

## 1. Introduction

Bone healing is critically dependent on the recruitment of skeletal stem cells and the re-establishment of a vascular network supplying the injured area [1–3]. Failure of either of these elements is likely to result in non-union defects. Reconstructive surgery with autologous bone tissue grafts is the preferred approach to treat such defects, however the limited volume of graft material available and risk of donor site morbidity makes this approach difficult [4–6]. In the absence of perfusion, the graft may fail due to avascular necrosis, and/or deficient integration of the graft with the adjacent host tissue. To address this challenge, contemporary tissue engineering approaches using cell-based and scaffold-assisted technologies have been developed but do not accommodate the need for immediate perfusion, thereby placing a modest upper limit on the size of defect that can be repaired. To circumvent this limitation, new perfusion-based model systems with both endothelial and osteogenic cells need to be developed to realize a scalable, vascularized bone graft in the long term.

Two-(2D) and three-dimensional (3D) cell cultures with endothelial cells encapsulated within hydrogels have shown a high potential for vasculature creation in in vitro settings [7]. These culture models are often supplemented with growth factors like vascular endothelial growth factor (VEGF), basic fibroblast growth factor (bFGF), or bone morphogenetic proteins (BMPs) to promote formation of vasculature and/or mineral deposition. However, this strategy not only is prohibitively expensive but also riddled with side effects such as unwanted tumor growth [8–11]. Alternatively, a growth factor free strategy of co-culturing endothelial and osteogenic cells within biomimetic hydrogels have been demonstrated to increase mineral deposition and vascularization [12–14]. Synergistic effects of such co-culture models are also often reported. Osteogenic cells have been reported to release VEGF, which in turn recruits endothelial cells and stimulates angiogenesis [14–16]. Conversely, pro-endothelial cells can release cytokines such as BMP-2 and endothelin-1, which support osteoblast differentiation and osteogenic mineralization [17–21]. Unfortunately, most co-culture models typically result in a disorganized network that cannot be readily perfused with nutrients [22–26].

To create an organized perfusable network within tissue constructs, several engineering methods such as needle-molding [27–31], sacrificial/fugitive-molding [32–34], lithography-based techniques [35] and direct [36,37] or indirect bioprinting [38–43] have been used. These strategies have demonstrated that the incorporation of perfusable channels within tissue constructs can yield enhanced cellular viabilities and these approaches can be potentially used to generate perfusion-based model systems for bone tissue engineering [44,45].

In this work, we have designed a fully customizable ‘plug-and-flow’ polycarbonate bioreactor containing 3D printed perfusable constructs. With this system, we have developed a co-culture model consisting of HUVECs and bone-like Saos-2 cells encapsulated within a Gelatin Methacrylate (GelMA)-collagen hydrogel for bone tissue engineering. The constructs, which contain two 500 μm inner diameter channels spaced 1 mm apart, were perfused with osteogenic media for up to two weeks in order to determine the independent and synergistic effects of co-encapsulating HUVECs and Saos-2 cells on mineral deposition in diffusion limited spaces.

## 2. Materials and methods

### 2.1. Fabrication of frames and bioreactor

Acrylonitrile butadiene styrene (ABS) frames with an inner size of 10 mm × 6 mm × 3 mm were designed and printed using a MakerBot 3D printer. The inner space of the ABS frame was divided into two 1 mm × 6 mm × 3 mm reservoirs for media to enter and exit as well as a 6 mm × 6 mm × 3 mm void between the reservoirs to house the cell-laden hydrogels. The center void contained two 1 mm holes on each side with 2 mm spacing in between for holding two parallel 500 μm diameter water soluble polyvinyl alcohol (PVA, eSUN) pipes that were also printed using a MakerBot 3D printer. The ABS frames and PVA pipes were UV sterilized overnight before adding the cell-laden hydrogels.

Polycarbonate bioreactors were fabricated using a MT300 ProCNC Milling Center. The bioreactor was composed of a base with four 13 mm × 9 mm × 3 mm chambers and a corresponding top piece. Each chamber had two vertical holes on both sides for inserting perfusion needles. Poly(dimethylsiloxane) (PDMS; 4:1 base:curing agent, Ellsworth Adhesives) was cast and cured around the chambers to ensure a seal between the polycarbonate base and top. The bioreactor was assembled using 10 screws prior to perfusion.

### 2.2. Preparation of GelMA-collagen blends

GelMA macromer was prepared by dissolving 8 ml of methacrylic anhydride (Sigma) in 100 ml of a 10% (w/v) porcine skin gelatin solution (Sigma) at 60 °C. The mixture was then diluted with 100 ml DPBS and dialyzed at 50 °C for 1 week before being lyophilized. 20% (w/v) GelMA pre-polymer solution with 0.25% (w/v) photo-initiator Irgacure 2959 (Specialty Chemicals) was prepared to be mixed with collagen. The GelMA-collagen prepolymer solution blend was prepared by first mixing 0.9 μl 1N NaOH with 7.2 μl of 10× Medium 199 (M199, Gibco). Next, 36 μl of 3 mg/ml collagen I rat tail solution (Gibco) was gently added to the NaOH/M199 solution and mixed thoroughly until the collagen was neutralized. Once the collagen was neutralized, 25.2 μl of 20% GelMA was added to the collagen mixture to generate the GelMA-collagen blend.

### 2.3. Cell encapsulation and perfusion

Human osteosarcoma cells (Saos-2, ATCC® HTB-85TM) and primary human umbilical vein endothelial cells (HUVECs, ATCC) were cultured and passaged at 37 °C with 5% CO_2_ according to a standard cell culture protocol. Dulbecco’s modification of eagle’s media (DMEM, Gibco) containing 1% penicillin-streptomycin (Gibco), 1% Glutamax (Gibco) and 10% fetal bovine serum (FBS, Atlanta Biologicals), and VascuLife VEGF-Mv endothelial complete kit (Lifeline Cell Technology) were used for culturing the Saos-2 and HUVEC cells respectively. Osteogenic media was prepared by adding 100 μM L-ascorbic acid-2-phosphate (AA2P, Sigma-Aldrich), 5 mM β-glycerophosphate (BGP, Sigma-Aldrich), and 10 nM dexamethasone (DEX, Sigma-Aldrich) with the complete DMEM to induce mineral formation during perfusion.

Prior to encapsulating cells in the GelMA-collagen blend, the Saos-2 and HUVEC cells were trypsinized and counted. For each construct either 10^6^ HUVECs, 10^6^ HUVECs + 5 × 10^5^ Saos-2, or 5 × 10^5^ Saos-2 were resuspended in 4.5 μl DMEM. The cell solution was mixed with 115.5 μl of GelMA-collagen prepolymer solution and pipetted gently into the center of the printed ABS frame. The cell-laden hydrogel was UV crosslinked using a LED controller (Hamamatsu Photonics K.K., Japan) at 5 mW/cm^2^ for 90 seconds. The constructs were incubated in DMEM overnight at 37 °C to dissolve the PVA pipes before being transferred to the polycarbonate bioreactor. The bioreactor was connected to a syringe pump (NE-300 Just InfusionTM, New Era) to perfuse osteogenic DMEM through each channel at a speed of 0.2 μl/h per channel for up to 2 weeks.

### 2.4. Cell viability

Cell-laden constructs were stained with Calcein-AM (1:2000 dilution, Life Technologies) and ethidium homodimer-1 (1:500 dilution, Life Technologies) for 45 minutes to test viability 1 day after encapsulation and 14 days after perfusion. The constructs were cut into 1 mm thick slices to image cross-sections of the gels. Both brightfield and fluorescence images were captured using an inverted fluorescent microscope (Zeiss Axiovert 40 CFL).

### 2.5. MicroCT

Perfused constructs were removed from the bioreactor and fixed in 4% formaldehyde for 24 hours prior to being imaged via microCT (microCT 40, Scanco Medical AG, Bruttisellen, Switzerland). Samples were placed lengthwise in a 16 mm diameter sample holder, kept hydrated with PBS, and imaged at a 16 μm isotropic voxel resolution (55 kV, 145 mA, 200 ms integration time). Mineralized tissue volume (BV) and density (BMD) was calculated by applying a lower global threshold of 166 mg HA cm^−3^ to images and those values were used to determine total mineral content. In order to calculate the total mineral content of the inner third of the constructs, the images were digitally contoured to isolate the areas of interest.

### 2.6. Histological and fluorescent staining

After microCT imaging, the cell-laden constructs were removed from the sample holders and immersed in a 30% sucrose solution overnight. The samples were then frozen using O.C.T. embedding medium (Tissue-Tek) before being completely sectioned into 10 μm thick slices with a Leica CM3050 cryostat (Leica Biosystems, Germany) and mounted on poly-lysine modified glass slides.

#### 2.6.1. Alizarin red staining

40 mM Alizarin red S solution at pH 4.1 (Sigma Aldrich) was applied to sections and incubated for 5 minutes before being washed with distilled water. The sections were dehydrated using 2 changes of 100% ethanol and xylene. The stained sections were mounted with Permount (Fisher Scientific) before imaging.

#### 2.6.2. Hematoxylin and eosin (H&E) staining

The sections were stained using a H&E staining kit (Vector laboratories). Hematoxylin solution was applied to slides and incubated for 5 minutes before being rinsed with distilled water for 30 seconds. The bluing reagent was added and incubated for 15 seconds. After washing slides with water for 30 seconds, the sections were immersed in 100% ethanol for 10 seconds. Eosin Y solution was applied to sections and incubated for 2 minutes. The sections were then rinsed and dehydrated with 3 changes of 100% ethanol and xylene and mounted with Permount prior to imaging.

#### 2.6.3. Osteocalcin (OCN) and CD31 staining

The slides were blocked with a 3% BSA (Sigma) solution for 30 minutes and incubated with OCN (1:50, Catalog MA143028, Invitrogen) and CD31 (1:50, Catalog PA579805, Invitrogen) primary antibodies at 4 °C overnight. The following day, the slides were incubated with the corresponding OCN (4 μg/ml, Alexa 568-labeled goat anti-mouse IgG1, Catalog A21124, Invitrogen) and CD31 (4 μg/ml, Alexa 488-labeled goat anti-rabbit IgG (H+L), Catalog A11034, Invitrogen) secondary antibodies and 0.2% BSA for 45 minutes. Images were taken using a Leica EZ4 W microscope for bright field microscopy and a Nikon Eclipse Ti microscope for immunofluorescence imaging. The images were stitched and processed using ImageJ.

### 2.7. Statistical analysis

MicroCT data was entered into Microsoft Excel to calculate the mean and standard deviation and Student’s t-tests were used to assess statistical significance of differences. P-values less than 0.05 were considered significant.

## 3. Results

### 3.1. Plug-and-flow bioreactor fabrication

**Figure 1:**
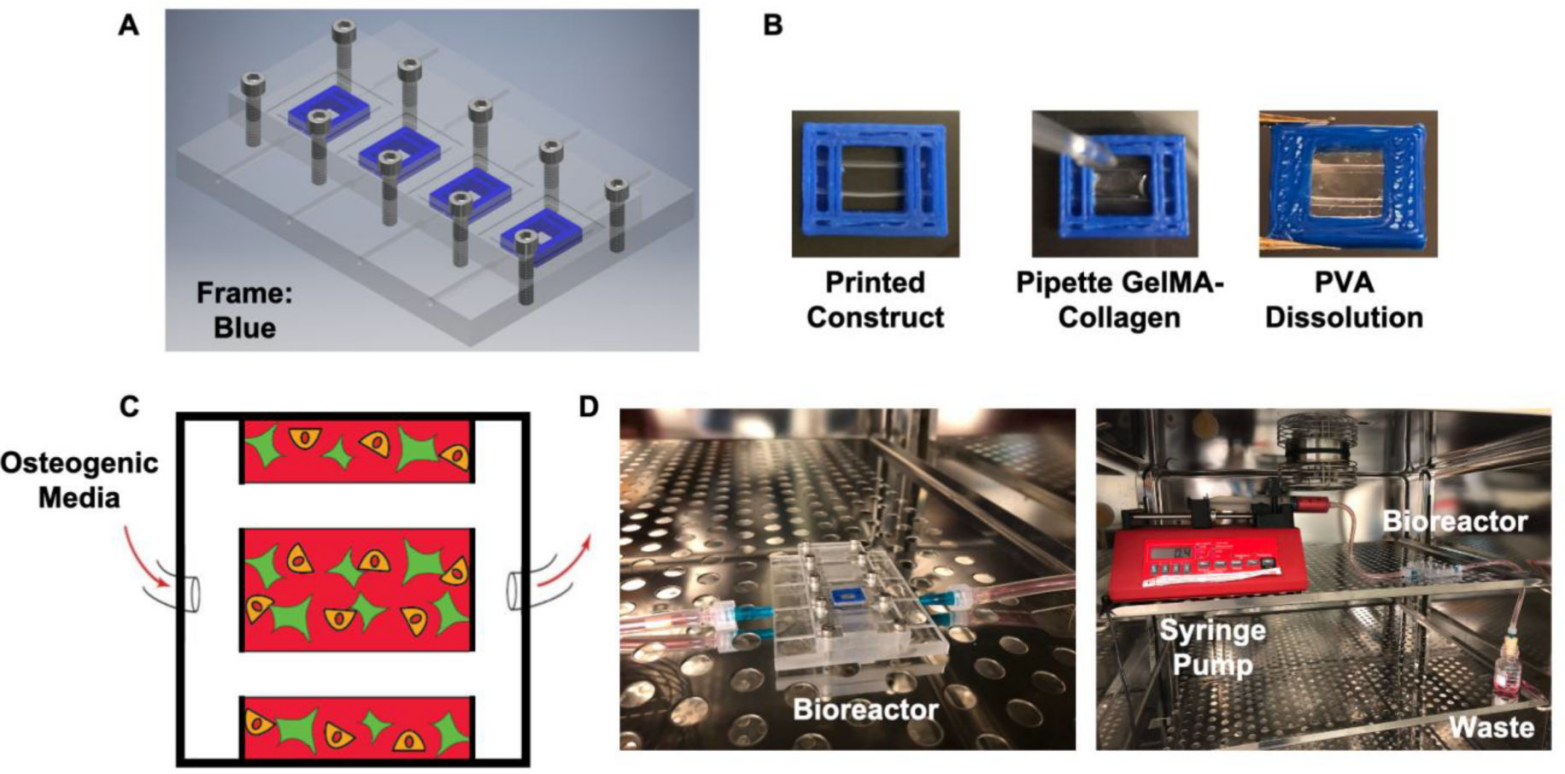
Design of 3D-printed construct and bioreactor setup. A schematic rendering of a two-piece polycarbonate bioreactor containing a PDMS gasket between layers (A). 3D printed ABS constructs containing two dissolvable PVA pipes (B, left) are designed to fit snuggly within the bioreactor for perfusion with osteogenic media (C). Prior to placement in the bioreactor, the GelMA-collagen blend containing cells is pipetted into the construct (B, middle), UV cured, and placed in culture media to dissolve the PVA pipes (B, right). After sealing the two-piece bioreactor, osteogenic media is perfused via a syringe pump in a sealed incubator (D).

Based on designs from previous work [46], a two-piece plug-and-flow bioreactor was machined from UV resistant polycarbonate in order to house acrylonitrile butadiene styrene (ABS) cages containing dual perfusable channels (Figure 1A). The mechanically supportive ABS cages were printed using a commercially available 3D printer along with sacrificial polyvinyl alcohol (PVA) pipes (Figure 1B, left) and were designed to be press fit into the polycarbonate bioreactor base. Prior to cellular incorporation, the bioreactor and 3D printed cages were sterilized using conventional methods.

After sterilization, the GelMA-collagen prepolymer solution containing either HUVECs (H), Saos-2 cells (S), or HUVECs plus Saos-2 cells (H+S) were cast into the cages and UV cured for 1 minute 30 seconds (Figure 1B, middle). PVA pipes were eluted from the constructs via immersion in cell culture media prior to being placed within the bioreactor base (Figure 1B, right). Once placed within the cell culture incubator, a syringe pump was used to deliver osteogenic media to the constructs for up to two weeks at a constant rate of 0.2 ml/hr per pipe (Figure 1D). Waste collection occurred in a receptacle below the bioreactor.

Prior to the incorporation of cells into this model system, preliminary experiments were preformed to determine whether or not the channels should be either lined with HUVECS, or if the HUVECS should be incorporated into the hydrogel matrix with the Saos-2 cells. Like other studies involving HUVECS, we had in previous work used static systems consisting of channels lined with HUVECs [44], but had not investigated how constant perfusion would impact the cells. To this end, before conducting our dual channel experiments, we employed a simple single pipe system consisting of either HUVEC lined channels with bone like cells contained in the bulk hydrogel, or a combination of HUVECS and bone like cells encapsulated together in the bulk matrix (Figure S1). After two weeks of perfusion with osteogenic media, it was shown via microCT that the HUVEC lined channels inhibited mineralization and that HUVECS contained in the bulk hydrogel with the Saos-2 cells resulted in a significantly increased amount of mineral deposition.

### 3.2. Cellular viability within plug-and-flow bioreactor

**Figure 2:**
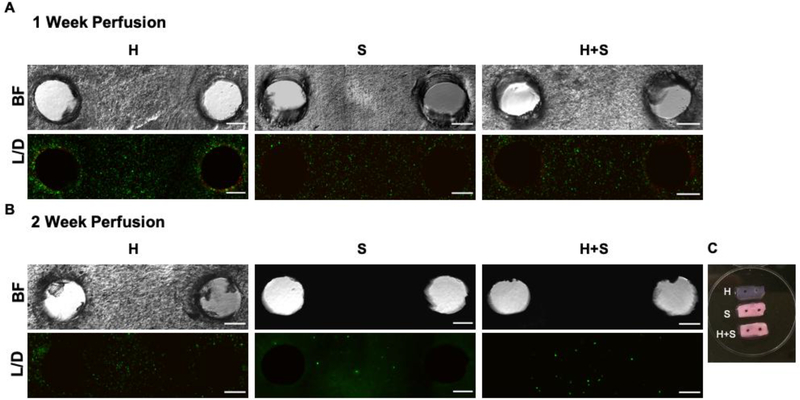
Cellular viability in perfused constructs. Cell viability (L/D) by fluorescent live (green)/dead (red) staining after either 1 week (A) or two weeks (B) of osteogenic perfusion (scale bar = 200 μm). Brightfield (BF) images of stained construct slices are opaque when mineral is present (B, S and H+S). Macro scaled view of H, S, and H+S slices show how mineral deposition changes the GelMA-collagen opaqueness (C). Images are representative of three independent 1 week and 2 week experiments.

Cellular viability was assessed one week after encapsulation and subsequent perfusion with osteogenic media (Figure 2A). To determine viability, the hydrogels were removed from the ABS cages and sliced into 1 mm slabs prior to being stained with Calcein and Ethidium Homodimer. Fluorescent imaging showed minimal cell death around the two dissolved pipes (red), indicating there was minimal process induced necrosis due to the encapsulation. Additionally, there was a high cell viability in all areas of the hydrogel, suggesting that the perfusion of nutrients was sufficient for cell survival (green).

After two weeks of perfusion with osteogenic media, a different set of constructs were assessed for viability to determine if the plug-and-flow system was suitable for long term perfusion studies (Figure 2B). Bright field images of the constructs showed robust mineralization in the samples containing osteogenic cells (S and H+S), and the unmineralized HUVEC only control showed high cell viability (green). Due to the opaqueness of the mineralized constructs, however, the viability of the S and H+S samples could not be assessed (Figure 2B,C).

### 3.3. Construct mineralization

**Figure 3:**
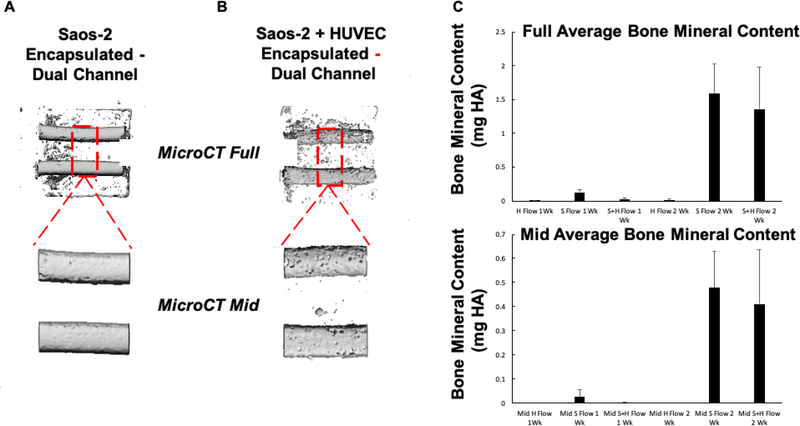
MicroCT analysis of mineral deposition. Representative full and middle third microCT images of the mineral deposited in the GelMA-collagen matrix by encapsulated Saos-2 cells after two weeks of osteogenic media perfusion (A). In constructs containing only Saos-2 cells, mineral was deposited predominately at the channel peripheries and not between the channels, as indicated by the inner third images. When HUVECS are added in a 2:1 ratio with Saos-2 cells, the Saos-2 cells deposited more mineral in the center of the constructs between the channels, as indicated by the representative full and middle third images (B). Quantification of mineral deposition in the whole microCT images and the isolated middle third images showed that there was no significant increase in total mineral deposition in co-cultured constructs as compared to Saos-2 constructs, indicating that the HUVECS facilitated mineralization between the channels as opposed to only the channel walls (n = 3 for HUVEC only samples, n = 5 for rest) (C).

MicroCT was used to evaluate the mineral formation in the entire construct (Full) as well as between the perfusable channels (Mid) after both one (1 Wk) and two (2 Wk) weeks of perfusion. From the full microCT scans, we observed that mineral formed around the entire length of the pipes in both the S and H+S samples, but no mineral was observed in the H only controls. Interestingly, while there was no difference in the total mineral content between the S and H+S constructs, mineral was found to be more dispersed throughout the center H+S hydrogel constructs than the S constructs, where mineral deposition was largely found adjacent to the perfused channels (Figure 3A–C, Top).

As we observed heavy mineralization adjacent to the inlet and outlet, we focused specifically on the inner third of the construct (Figure 3A,B,bottom). Similar to the quantification of the full constructs, at both one and two weeks there was not a significant difference in the amount of mineral produced between the S and H+S constructs (Figure 3C), however imaging showed that the S constructs contained robust mineralization along the channel wall while H+S constructs tended to mineralize both around and between the channels.

### 3.4. Histological analysis and fluorescent staining

**Figure 4:**
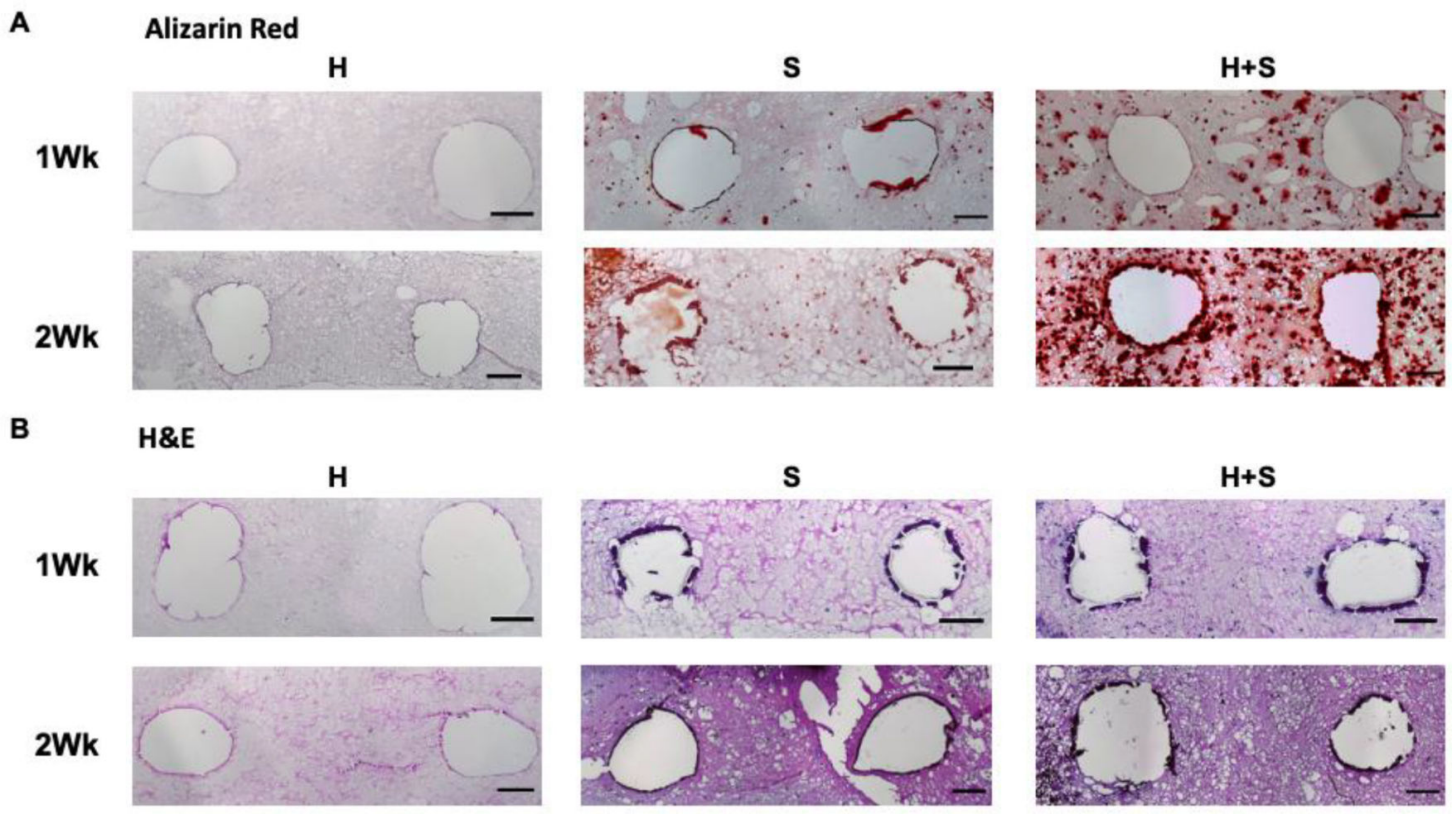
Histology of perfused constructs. Representative Alizarin Red (A) and H&E (B) histology stains of constructs after one or two weeks of perfusion with osteogenic media (scale bar = 200 μm). HUVEC only samples (H) showed no calcium mineral deposition after two weeks of perfusion, Saos-2 only samples (S) showed mineralization mainly along the channel peripheries, and HUVEC+Saos-2 samples (H+S) showed mineralization throughout the entirety of the construct.

Alizarin red S staining was used to visualize the distribution of deposited mineral in the H, S, and H+S samples after both one and two weeks of perfusion to confirm that the mineral deposition observed by microCT was composed of calcium (Figure 4A). After both one and two weeks of perfusion, Alizarin-stained mineral was found to accumulate around the channels of S and H+S constructs, whereas no mineralization was observed in H only constructs. Furthermore, a greater degree of cell-associated mineralization was observed in the space between pipes of the H+S constructs than the S only constructs suggesting that the HUVECs aided mineralization of the hydrogel in areas away from the perfused media.

H&E staining was used to visualize the differences in construct cellularity over time (Figure 4B). After one week of perfusion, the S and H+S constructs were similar, however more staining was observed away from the channels of the H+S hydrogels, indicating more cells were located further away from the perfused nutrient supply. After two weeks of perfusion a similar result was observed.

**Figure 5:**
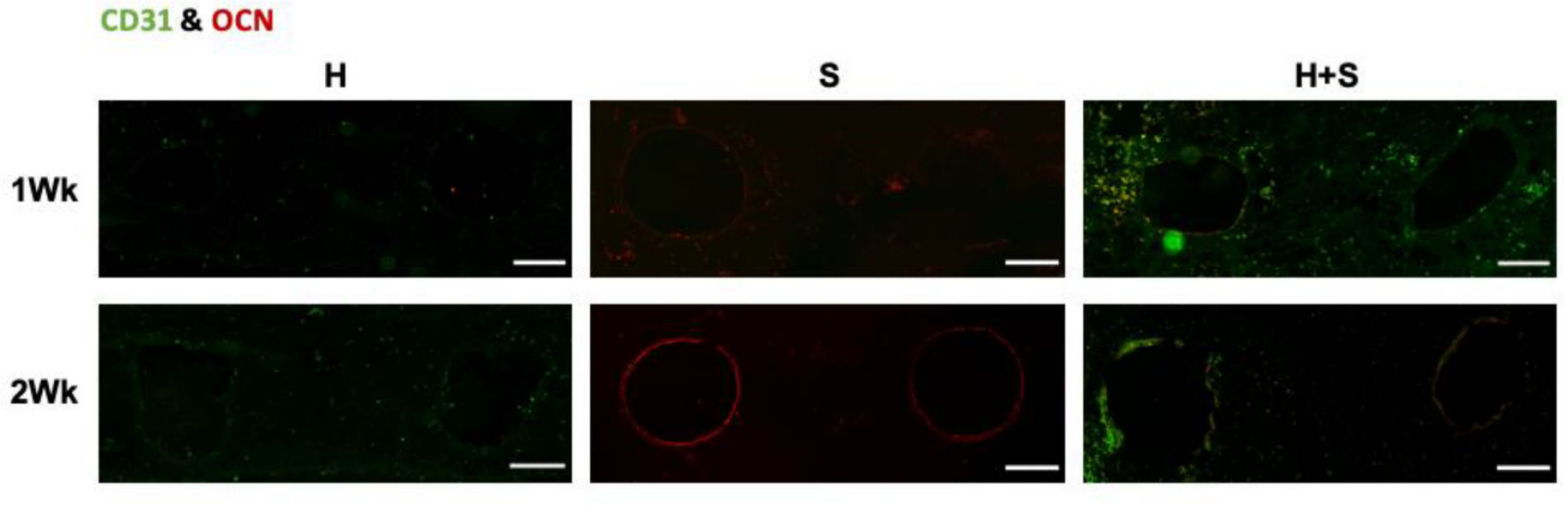
CD31 and OCN immunofluorescent staining. Representative CD31 and OCN immunofluorescence images of constructs after one or two weeks of perfusion with osteogenic media. HUVEC only samples (H) showed positive CD31 staining with no OCN positive cells, while Saos-2 only samples (S) showed positive OCN staining with no CD31 positive cells. Co-cultured samples (H+S) were positive for both CD31 and OCN, however staining after two weeks of perfusion was inhibited by the sample opaqueness caused by deposited mineral (scale bar = 200 μm).

Platelet endothelial cell adhesion molecule (CD31), a protein found on human endothelial cells, and osteocalcin (OCN), a protein found in bone, were immunofluorescently stained in histological sections to further investigate how the endothelial cells impacted the mineral deposition within the perfused constructs (Figure 5). After both one and two weeks of perfusion, the H only controls showed only CD31 positive staining, as expected. Similarly, the S only constructs showed minimal OCN staining after one week of perfusion, while after two weeks OCN was prevalent around the channel peripheries and was generally absent between the channels. The H+S samples showed both CD31 and OCN staining at both week 1 and week 2, however the CD31 staining decreased after two weeks of perfusion both around and between the channels, indicating that the mineral deposition was interfering with the fluorescent imaging.

## 4. Discussion

In the past, strategies for creating a bone tissue engineered construct have normally revolved around the creation of a hard bio-ceramic scaffold made of materials such as calcium phosphate or hydroxyapatite followed by the seeding of cells on them post-manufacturing. Unfortunately, these approaches are often plagued by non-homogenous cell distribution and limited nutrient diffusion, ultimately resulting in necrotic cores and osteogenesis restricted to construct surfaces [15,47–50]. Although the rationale of these strategies has been to mimic the mechanical and material properties of the mineralized bone matrix, it is important that a complete tissue engineering solution also address the complex biological organization of living bone tissue. Not only should the construct be comprised of a material capable of supporting the numerous cell interactions required for proper bone growth, but it must also contain a defined perfusable network that is able to properly deliver nutrients and systemic signaling molecules to bone cells [51–55].

In our study, we combined a soft, GelMA-collagen hydrogel containing user defined perfusable channels with a structurally supportive ABS cage. While the cage provided the necessary structural support for the model bone tissue system, the soft hydrogel and perfusable network served to overcome the limitations found in machined, hard scaffolds. In our model system, mineral deposition was observed throughout the entirety of the scaffold and cell viability was maintained through two weeks of perfusion.

Previous reports of tissue engineered constructs involving co-cultures of endothelial cells with other stromal/parenchymal cells have depended upon a two-step process to establish a confluent endothelial lining along the channel lumens that allows the selective flow of nutrients into the material [44,45]. Initially, a similar two-step process was used for this study with Saos-2 cells encapsulated within the GelMA matrix followed by static seeding of HUVECs such that they lined the channel (Figure S1). We found that after two weeks of perfusion with osteogenic media, HUVEC lined channels inhibited mineral formation. In contrast, co-encapsulating HUVEC and Soas-2 cells within GelMA in a single step resulted in enhanced mineral deposition within the GelMA. To this end, this co-encapsulation strategy has also shown promise in the field of tissue engineering by other groups. In one instance, encapsulated HUVECS within collagen gels rearranged into cords over the course of only 10 hours [56], while HUVECS embedded within a fibrin gel containing supportive fibroblasts showed capillary formation between two larger, perfusable channels [57].

The addition of HUVECS alongside the Saos-2 cells in the bulk matrix resulted in the deposition of mineral within the center of our constructs between the two perfusable channels. Previous studies with Saos-2 cells encapsulated within GelMA hydrogels have shown that there is a diffusion limitation at the center of the hydrogel that inhibits mineral deposition [58,59]. This previously observed inhibition is reinforced by observation of in vivo tissue architecture that shows most cells reside within 100–200 μm of a nutrient supply, which allows for normal physiologic function [15,60]. While mineral was observed consistently along the channel walls, only constructs that contained both Saos-2 and HUVECS deposited a significant amount of mineral in the space between the two perfused channels.

## 5. Conclusions

This study demonstrated that our model system could be used to study directed bone mineralization and the synergistic response between endothelial and bone-like cells. Based on our results, not only can user-defined channels be used to control the location of mineral deposition within a thick, tissue like construct, the addition of endothelial cells to the system appears to enable mineral deposition in otherwise diffusion limited spaces. While the mechanism of how the endothelial cells facilitated the mineralization between the perfusable channels is not clear, this work provides a template for studying engineered bone tissue substitutes.

## Supplementary Material

Figure S1
