# Supplementary material for: Perfusion-based co-culture model system for bone tissue engineering
Source: AIMS Bioeng. Author manuscript; Available in PMC 2020 Nov 5. (PMC7643915; doi:10.3934/bioeng.2020009)
Supplement: Figure S1 [file NIHMS1639737-supplement-Figure_S1.docx]

**Supplementary Figures**


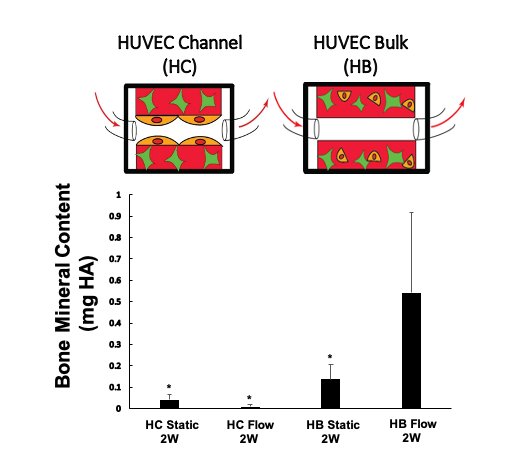


**Supplementary Figure 1: MicroCT analysis of mineral deposition within a co-culture single pipe system.** HUVECS were either seeded along the perfusable channels (HC) or added in a 2:1 ratio with Saos-2 cells in the bulk GelMA matrix (HB) and the constructs were either perfused with (Flow) or left in static control wells containing (Static) osteogenic media for two weeks. After two weeks, HUVECS encapsulated in the bulk material significantly increased Saos-2 mineral deposition as compared to the constructs containing HUVEC lined channels and their static controls (* p<0.05 as compared to HB Flow 2Wk, n=5).
